# Near‐IR to Near‐IR Upconversion Luminescence in Molecular Chromium Ytterbium Salts

**DOI:** 10.1002/anie.202007200

**Published:** 2020-08-18

**Authors:** Jens Kalmbach, Cui Wang, Yi You, Christoph Förster, Hartmut Schubert, Katja Heinze, Ute Resch‐Genger, Michael Seitz

**Affiliations:** ^1^ Institute of Inorganic Chemistry University of Tübingen Auf der Morgenstelle 18 72076 Tübingen Germany; ^2^ Division Biophotonics Federal Institute for Materials Research and Testing (BAM) Richard-Willstätter-Strasse 11 12489 Berlin Germany; ^3^ Institute of Chemistry and Biochemistry Freie Universität Berlin Arnimallee 22 14195 Berlin Germany; ^4^ Department of Chemistry Johannes Gutenberg University of Mainz Duesbergweg 10–14 55128 Mainz Germany

**Keywords:** chromium, energy transfer, luminescence, upconversion, ytterbium

## Abstract

Upconversion photoluminescence in hetero‐oligonuclear metal complex architectures featuring organic ligands is an interesting but still rarely observed phenomenon, despite its great potential from a basic research and application perspective. In this context, a new photonic material consisting of molecular chromium(III) and ytterbium(III) complex ions was developed that exhibits excitation‐power density‐dependent cooperative sensitization of the chromium‐centered ^2^E/^2^T_1_ phosphorescence at approximately 775 nm after excitation of the ytterbium band ^2^F_7/2_→^2^F_5/2_ at approximately 980 nm in the solid state at ambient temperature. The upconversion process is insensitive to atmospheric oxygen and can be observed in the presence of water molecules in the crystal lattice.

## Graphical abstract

Strong upconversion luminescence at 780 nm can be observed under ambient conditions after photoexcitation at 980 nm in a new ionic solid consisting of discrete Yb^III^‐ and Cr^III^‐complexes. The upconversion process is insensitive to atmospheric oxygen and can be observed in the presence of water molecules in the crystal lattice.

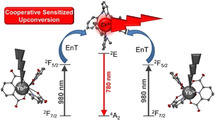

## Introduction

Metal‐based upconversion (UC) transforming low‐energy photons into an anti‐Stokes‐shifted luminescence is a very attractive non‐linear process for fundamental studies as well as for future applications. Examples are solid inorganic host matrices with low‐phonon energies doped with transition metal or lanthanoid cations, either as bulk materials^1^ or, more recently, as nanocrystalline systems.^2^ UC was long considered to be impossible in discrete metal‐organic complexes^3^ due to the pronounced non‐radiative deactivation of the excited metal states by high‐frequency oscillators present in organic ligands like ‐OH, ‐NH or ‐CH groups.^4^ In the past few years, however, many advances have been achieved in implementing metal‐based UC in molecular complex species, some even at ambient temperature and in solution.^5^ This includes metal chelate‐organic chromophore combinations,^6^ mononuclear metal complexes,^7^ and hetero‐oligometallic sensitizer–activator architectures.^8^, ^9^ The latter have shown to hold the greatest potential for efficient UC, especially for energy transfer upconversion (ETU) but also for cooperatively sensitized upconversion (CSU). For both UC schemes, sensitizer metal centers (S) with appropriate energy levels and sufficiently long luminescence lifetime are necessary to successfully populate an activator (A) excited state with approximately twice the energy of the excited sensitizer state at relatively low excitation power densities. Among the best sensitizing metal centers are Yb^3+^ (^2^F_5/2_ at ≈10 250 cm^−1^, ≈976 nm) and Cr^3+^ (octahedral geometry: ^2^E/^2^T_1_ at ≈15 000‐12 400 cm^−1^, ≈665–805 nm depending on the ligand field). This has been demonstrated for several emissive UC activators in molecular systems, for example the lanthanoids Er^3+^ and Tb^3+^.^8^, ^9^ The earth‐abundant metal Cr^3+^ has also gained renewed interest as downshifting luminophore/sensitizer,^10^ on one hand because of the recently developed class of “molecular ruby” emitters which show very high luminescence quantum yields of the ^2^E/^2^T_1_ phosphorescence of up to 30 % at room temperature in solution in the absence of oxygen,^11^ and on the other hand as successful antenna moieties for the downshifting sensitization of near‐IR lanthanoid luminescence.^12^, ^13^

**Figure 1:**
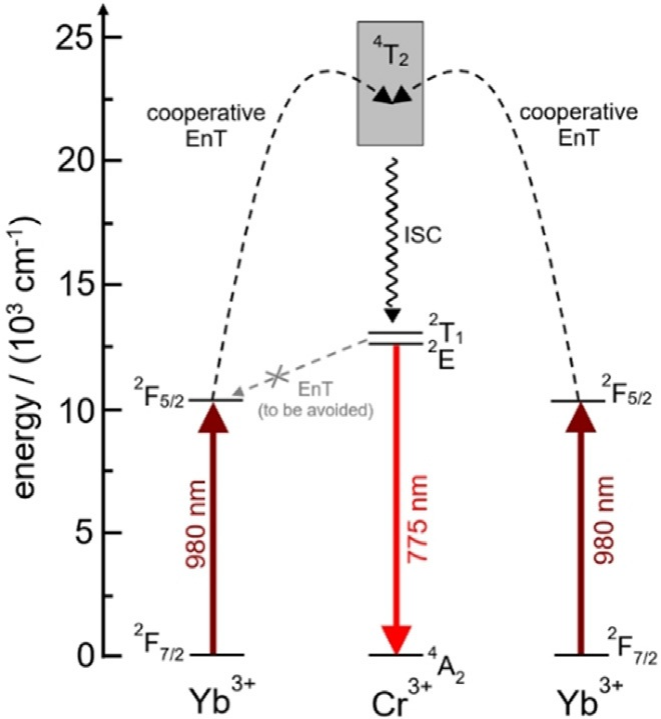
Schematic partial energy‐level diagram of the energy levels of Yb^3+^ and Cr^3+^ (energies given for *mer*‐[Cr(ddpd)_2_]^3+^) relevant for cooperatively sensitized UC involving two Yb^3+^ centers absorbing 980 nm light and sensitizing the emission of the Cr^3+^ activator.

Two decades ago, Güdel et al. reported an interesting UC Scheme for the generation of ^2^E UC emission for solid state hosts such as Y_3_Ga_5_O_12_ co‐doped with Yb^3+^ as sensitizer and Cr^3+^ as activator.^14^ These compounds operate via CSU where two excited Yb^3+^ cooperatively transfer the energy from their ^2^F_5/2_ states to an excited quartet state of Cr^3+^ (^4^T_2_/^4^T_1_) which subsequently populates the emissive ^2^E state by intersystem crossing (ISC) (Figure 1). This Scheme is particularly interesting because both, excitation and UC emission, are in the near‐IR spectral window, increasingly used for bioimaging.^15^ In molecular systems, near‐IR to near‐IR upconversion is unknown and the few systems utilizing the couple Yb/Cr reported so far exhibited UC only at very low temperatures (usually below 100 K) in extended solid inorganic matrices. In the past, however, reports on efficient downshifting energy transfer (EnT) ^2^E(Cr^3+^)→^2^F_5/2_(Yb^3+^),^12^, ^13^ that led to deactivation of the UC‐emissive ^2^E state, made the successful implementation of this attractive UC Scheme unlikely. Especially Cr^3+^/Yb^3+^‐architectures with highly efficient Dexter EnT (here total angular momentum allowed for Δ*J*=1)^16^ in hexacyanidochromate‐ and oxalato‐bridged coordination compounds13a, 13b, 13c seemed unsuitable for this purpose. On the other hand, dipole‐dipole EnT (Förster) Cr^3+^→Yb^3+^ in oligometallic molecular systems also showed unfavorably high EnT efficiencies of up to ca. 50 % despite being forbidden by the total angular momentum selection rule (Δ*J*=2,4,6).13a, 16

With these challenges of the Cr^3+^/Yb^3+^ pair in mind, we revisited the design concept for molecular Yb‐Cr‐UC. This led to a new photonic material composed of easily accessible Cr^3+^ and Yb^3+^ complex ions which shows ^2^E/^2^T_1_ UC at room temperature already at relatively low excitation power densities.

## Results and Discussion

**Figure 2:**
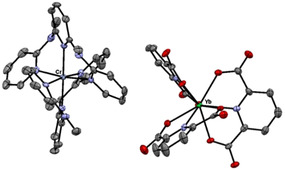
Thermal ellipsoid plot of the asymmetric unit in **3‐Yb** (Ortep 3 for Windows,^20^ 50 % probability level). Lattice solvent molecules and hydrogen atoms are omitted for clarity.

**Scheme 1:**
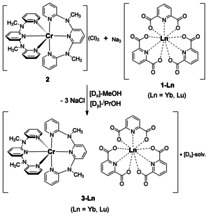
Synthesis of the chromium‐lanthanoid salts **3‐Ln**.

The main idea was to avoid Dexter EnT from ^2^E(Cr^3+^) to ^2^F_5/2_(Yb^3+^) and opt for a system, where Cr^3+^→Yb^3+^ EnT was only possible by a less efficient Förster mechanism. Therefore, we utilized spatially separated metal centers in discrete coordination environments. For the realization of this design, we chose the complex *mer*‐[Cr(ddpd)_2_]^3+^ (ddpd=*N*,*N*′‐dimethyl‐*N*,*N*′‐dipyridine‐2‐ylpyridine‐2,6‐diamine). This Cr^3+^ complex shows a very high phosphorescence quantum yield *Φ* of up to 30 % in argon‐saturated CD_3_CN solution at room temperature (298 K) and even remains quite emissive in air‐saturated water with *Φ*=2.1 %.11c, 11d Despite earlier reports on the complex [Yb(dpa)_3_]^3−^ (dpa=2,6‐pyridine‐dicarboxylate) and the only moderately long lifetime of its excited ^2^F_5/2_ energy level in the solid state (solid **1‐Yb** at 295 K: *τ*=2.9 μs),^3^, 13c we chose this anion as counterpart for the Cr^3+^ complex because of its straightforward synthetic accessibility and its good match with [Cr(ddpd)_2_]^3+^ in terms of comparable size and opposite charge. The latter parameters were expected to facilitate the crystallization of the desired Cr/Yb ionic solid, where only intermolecular π‐π‐stacking interactions between the different ions occurs. The synthesis of our novel photonic material **3‐Yb** was achieved by mixing Na_3_[Yb(dpa)_3_]⋅6 H_2_O (**1‐Yb**)^17^ with [Cr(ddpd)_2_]Cl_3_ (**2**, see SI for details) in an alcoholic solution (Scheme 1). We also prepared the reference compound **3‐Lu** as a structural analogue of **3‐Yb**, thereby utilizing the photoinactive nature of Lu^3+^ with its 4f^14^ electronic configuration. **3‐Yb** and **3‐Lu** were obtained as bright yellow solids in good to excellent yields (57–88 %). Both, complex anion and cation,^18^ are chiral but were used as racemates. Elemental analysis of both compounds revealed large amounts of lattice water and methanol in the material (see SI for details). To suppress potentially severe non‐radiative deactivation of both the ^2^E/^2^T_1_ and ^2^F_5/2_ excited states via multiphonon relaxation by C‐H and O‐H oscillators,^4^ the syntheses were also repeated with [D_4_]‐MeOH/[D_8_]‐^i^PrOH. The X‐ray structural analysis of single crystals of **3‐Ln** grown from MeOH/^i^PrOH mixtures confirmed that all salts are isostructural, racemic mixtures of the complex ions (Figure 2, see also Table S1 and Figure S1 in the SI).^19^

**Figure 3:**
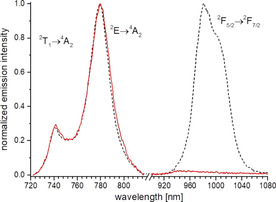
Normalized steady‐state emission spectra (λ_exc_=435 nm) of **3‐Yb** (dashed black line) and **3‐Lu** (solid red line) in the solid state at *T*=298 K in air. Excitation was at 435 nm. The relative intensities of the emission spectra of the different Cr^3+^ and Yb^3+^ emission bands were not comparable.

As intended, in the solid material, downshifting EnT in **3‐Yb** should only be possible by the forbidden Förster mechanism. In our crystal, each Cr^3+^ activator is surrounded by five Yb^3+^ sensitizers as nearest neighbors with a distance distribution of 8.75 Å< r_Cr‐Yb_ <9.07 Å (Figure S2 in the SI). Taking into account the distance relationship for S→A EnT (*k*_EnT_∝*r* ^−6^) and assuming similar contributions from all other parameters (e.g. orientation of the chromophores, dipole moments etc.), similar energy transfer rates to the central Cr^3+^ activator were expected for the five nearest sensitizers that should hence only vary by a factor of up to (8.75/9.07)^−6^=1.24. Selective excitation of **3‐Ln** at λ_exc_=435 nm into the ^4^A_2_→^4^T_2_ band11c, 11d of [Cr(ddpd)_2_]^3+^ produces the expected chromium phosphorescence ^2^E/^2^T_1_ with an emission maximum around 780 nm. For **3‐Yb**, excitation at 435 nm leads not only to the Cr^3+^ emission (Figure 3) but also to the appearance of a Yb^3+^ luminescence (^2^F_5/2_→^2^F_7/2_) at around 1000 nm (Figure 3). Since the chromium‐free precursor **1‐Yb** is not emissive upon excitation at 435 nm (Figure S3), this clearly indicated undesired Cr→Yb EnT in **3‐Yb**. Further evidence for a downshifting EnT between Cr^3+^ and Yb^3+^ was obtained by time‐resolved luminescence measurements under the same conditions (Table 1).

**Table 1:** Luminescence lifetimes τ and quantum yields Φ of compounds **3‐Ln** (Ln=Yb, Lu) in the solid state at 298 K for excitation at 435 nm.

Species	$\tau_{{}^{2}E\to {{}^{4}A}_{2}}^{\mathrm{air}}$	$\tau_{{}^{2}E\to {{}^{4}A}_{2}}^{\mathrm{argon}}$	$\tau_{{{}^{2}F}_{5/2}\to {{}^{2}F}_{7/2}}^{\mathrm{air}}$	$\Phi_{{}^{2}E\to {{}^{4}A}_{2}}^{\mathrm{air}}$
	(783 nm) [μs]^[a]^	(783 nm) [μs]^[a]^	(980 nm) [μs]^[a]^	[%]^[b]^
**3‐Yb**	390 (100 %)	380 (100 %)	9 (rise, 2 %) 369 (decay, 102 %)	5.9
				
**3‐Yb** (deut.)	160 (15 %) 390 (85 %)	180 (12 %) 370 (88 %)	12 (rise, 3 %) 373 (decay, 103 %)	5.8
				
**3‐Lu** (deut.)	280 (11 %) 660 (89 %)	320 (12 %) 720 (88 %)	n.a.	6.8

[a] Lifetimes are fitted mono‐ or biexponentially, percentages in parentheses give relative amplitudes of the components, estimated uncertainty of τ ±5 %. [b] Measured using an integrating sphere setup Quantaurus‐QY C11347‐11 (see Supporting Information for details), estimated uncertainty ±5 %.

The decay curve of the Cr^3+^‐centered ^2^E/^2^T_1_ emission of **3‐Yb** exhibited monoexponential decay kinetics with a long lifetime *τ*=390 μs. The decay profile of the Yb^3+^ emission revealed biexponential kinetics with a long luminescence lifetime of 369 μs, uncharacteristic for molecular Yb^3+^ species^4^ which normally show luminescence lifetimes in the low μs‐range. The lifetime of 369 μs closely matches the ^2^E/^2^T_1_ lifetime of the Cr^3+^ emission of 390 μs. In addition, a noticeable rise time component (*τ*=9 μs) was present. These observations are all typical for EnT from the long‐lived ^2^E state to Yb^3+^.13d, 13e, 13f As detailed before, this EnT could reduce the efficiency of the ^2^E upconversion luminescence by non‐radiatively depopulating this state. To quantify the potential loss in efficiency, we determined the quantum yield of the ^2^E/^2^T_1_ phosphorescence of **3‐Yb** and **3‐Lu** upon excitation at 435 nm (Table 1). These measurements yielded Φ values of 6.8 % and 5.8 % for deuterated **3‐Lu** and **3‐Yb**, respectively, and hence revealed only a moderate decrease of 15 % ^2^E quantum yield for **3‐Yb** relative to **3‐Lu**. This is favorably low compared to the loss due to Förster EnT reported for analogous downshifting systems in the literature (ca. 20–50 %),13d, 13e, 13f especially when considering that in our case each Cr^3+^ has considerably more next Yb^3+^ neighbors (here 5, previously 1 at similar distances r_Cr‐Yb_) as EnT acceptors. Surprisingly, neither the crystallization of **3‐Yb** from deuterated solvents nor the presence of oxygen significantly affected the luminescence decay kinetics of Cr^3+^ in **3‐Yb** and **3‐Lu** (Table 1). The decay profile of the Cr^3+ 2^E/^2^T_1_ emission in deuterated **3‐Lu** in air also showed biexponential decay kinetics and revealed considerably longer lifetimes than observed for **3‐Yb** (Table 1. Deuterated **3‐Lu**: τ_1_=660 μs, 89 % and τ_2_=280 μs, 11 %).

**Figure 4:**
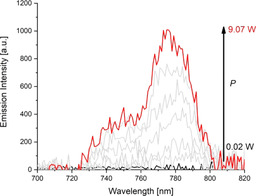
Excitation power‐density (*P*) dependence of the UC luminescence (^2^E/^2^T_1_→^4^A) of **3‐Yb** (298 K, solid, air) for Yb^3+^ excitation at λ_ex_=976 nm.

**Figure 5:**
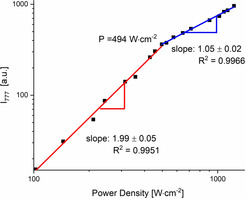
Log–log plot of the upconversion ^2^E luminescence (λ_em_=777 nm) versus the incident power density in **3‐Yb** (λ_ex_=976 nm, 298 K, solid)—gradients obtained by linear fitting for the low (red) and high (blue) power density regimes.

Finally, UC measurements of **3‐Yb** and **3‐Lu** were performed at 298 K under ambient atmosphere. Expectedly, **3‐Lu** did not yield any UC emission upon excitation at 976 nm. In contrast, excitation of the Yb^3+^ sensitizers in **3‐Yb** produced intense ^2^E/^2^T_1_ UC emission of the activator Cr^3+^ with a maximum around λ_em_=780 nm (Figure 4). Time‐resolved studies confirmed successful UC in **3‐Yb** and deuterated **3‐Yb**, while no luminescence signal was observed for **3‐Lu** (Figure S9). For **3‐Yb**, excitation power densities (*P*) as low as *P*≈67 W cm^−2^ were sufficient for the observation of UC which is a reasonably low threshold for UC by a normally not very efficient CSU mechanism.^5^ The *P* dependence of the UC emission intensity depicted in Figure 5 shows two distinct regions. Below *P*≈494 W cm^−2^, the number of excited Yb^3+^ is low and UC depends quadratically on *P* indicating a biphotonic process (log‐log plot: slope or photonic order of 1.99). At higher *P*, sensitizer saturation slowly occurs as indicated by a photonic order below 2 which eventually approaches 1 as is typical for a one‐photon process (slope or photonic order of 1.05).^21^

## Conclusion

In conclusion, by carefully revisiting earlier downshifting Cr^3+^/Yb^3+^ systems, we realized a novel near‐IR to near‐IR upconversion (UC) material by simply combining Cr^3+^ and Yb^3+^ complexes in an ionic solid. This expands the small number of molecular UC examples by a new pair of sensitizer/activator metal complexes. Importantly, UC can be realized with synthetically easily accessible non‐deuterated/non‐halogenated building blocks at room temperature in the presence of oxygen and water molecules. This proof‐of‐concept study will pave the way to a new class of photonic materials and enable new possibilities for the field of molecular UC.

## Conflict of interest

The authors declare no conflict of interest.

## Supporting information

As a service to our authors and readers, this journal provides supporting information supplied by the authors. Such materials are peer reviewed and may be re‐organized for online delivery, but are not copy‐edited or typeset. Technical support issues arising from supporting information (other than missing files) should be addressed to the authors.

Supplementary

Supplementary
