# Supplementary material for: Near‐IR to Near‐IR Upconversion Luminescence in Molecular Chromium Ytterbium Salts
Source: Angew Chem Int Ed Engl. 2020 Aug 18;59(42):18804–8. doi: 10.1002/anie.202007200 (PMC7589230; doi:10.1002/anie.202007200)
Supplement: Supplementary file 2 — Supplementary [file ANIE-59-18804-s002.pdf]

Angewandte Chemie
Eine Zeitschrift der Gesellschaft Deutscher Chemiker

Supporting Information

Near-IR to Near-IR Upconversion Luminescence in Molecular Chromium Ytterbium Salts

Jens Kalmbach[+], Cui Wang[+], Yi You, Christoph Förster, Hartmut Schubert, Katja Heinze, Ute Resch-Genger,* and Michael Seitz**

anie_202007200_sm_miscellaneous_information.pdf
anie_202007200_sm_cif.zip

Supporting Information

Table of Contents

1.	Materials / Methods	S2
2.	X-ray Single Crystal Structure Analysis	S4
3.	Luminescence Measurements	S6
3.1	Instruments	S6
3.2	Steady-State and Time-Resolved Luminescence Measurements	S8
3.3	Upconversion Measurements	S11
4.	References	S13

1. Materials / Methods

General Information

Acetonitrile (HPLC grade) and methanol (HPLC grade) were purchased by commercial suppliers and used without further purification. $Na_3[Ln(dpa)_3] \cdot x\ H_2O$[S1] (**1-Ln**, Ln = Yb, Lu, dpa = 2,6-dipicolinate) and ligand ddpd[S2] were synthesized in-house according to a literature procedure. Elemental analyses were performed by the Analytical Facility of the Institute of Inorganic Chemistry (University of Tübingen) using a Vario MICRO EL analyser. ESI mass spectrometry was performed on a Bruker Daltonics Esquire 6000 mass spectrometer by the central analytical facility of the Department of Chemistry (University of Tübingen).

Syntheses

Synthesis of $[Cr(ddpd)_2]Cl_3$ (2)

The ligand ddpd[S2] (500 mg, 1.72 mmol) was dissolved in methanol and the solution was deoxygenated with argon. Solid $CrCl_2$ (105 mg, 0.85 mmol) was added under inert conditions and the resulting solution was stirred for 12 h giving a green solution. After opening the flask to ambient conditions, the solution turned yellow and some dark material precipitated. The dark precipitate was removed by filtration over celite. Gaseous hydrochloric acid was slowly bubbled into the solution for 15 min. The solvent was removed under reduced pressure and the yellow solid was dissolved in a minimum amount of acetonitrile. This solution was layered with diethylether. Over 12 h at –25°C, a fine yellow solid precipitated. The product was collected by filtration and washed with diethylether. From the mother liquor, a further crop of material can be obtained by layering again with diethylether and storing at –25°C. Larger crystals can be obtained by diffusion of diethylether into a concentrated acetonitrile solution.

MS (ESI, pos. mode): *m/z* (%) = 211.37 (100, $[Cr(ddpd)_2]^{3+}$), 291.12 (20, $\{ddpd+H\}^+$), 334.57 (11, $\{[Cr(ddpd)_2]+Cl\}^{2+}$).

Optical properties in solution (λ_{exc} = 435 nm, λ_{em} = 775 nm):

$\Phi(H_2O, air)$ = 1.93 %, $\tau(H_2O, air)$ = 198 µs

$\Phi(H_2O, Ar)$ = 8.30 %, $\tau(H_2O, air)$ = 793 µs

$\Phi(CH_3CN, air)$ = 0.50 %, $\tau(CH_3CN, air)$ = 21 µs

$\Phi(CH_3CN, Ar)$ = 6.85 %, $\tau(CH_3CN, Ar)$ = 535 µs

3-Ln (Ln = Yb, Lu)

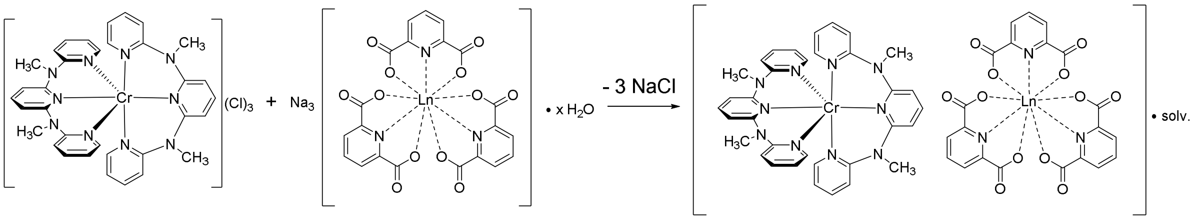

2 **1-Ln** Ln = Yb, Lu **3-Ln** Ln = Yb, Lu

General procedure:

In a 10 ml screw cap vial, $Na_3[Ln(dpa)_3] \cdot x\ H_2O$[S1] (**1-Ln**, 1.0 equiv., Ln = Yb: x = 6; Ln = Lu: x = 8) and $[Cr(ddpd)_2]Cl_3$ (**2**, 1.0 equiv.) were suspended in methanol ($[D_0]/[D_4]$). The yellow mixture was stirred until all components were dissolved. After stirring for additional 30 minutes at room temperature, the solution was layered with isopropanol ($[D_0]/[D_8]$, 2 mL). Upon standing for 1 to 3 days, large orange crystals of **3-Ln** were obtained which were also suitable for X-ray crystallographic analysis. The crystals were collected, washed with isopropanol ($[D_0]/[D_8]$, 3 × 1 ml), ice-cold methanol ($[D_0]/[D_4]$, 1 × 2 ml), and finally dried under reduced pressure. The product was obtained as yellow solid.

3-Yb:

$Na_3[Yb(dpa)_3] \cdot 6\ H_2O$ (33 mg, 39 mmol) and $[Cr(ddpd)_2]Cl_3$ (29 mg, 39 mmol) in MeOH (4 mL), precipitation with ^{i}PrOH → Yield: 45 mg (34 mmol, 88%).

Anal. Calcd. for: $C_{55}H_{43}CrN_{13}O_{12}Yb \cdot 5\ CH_3OH \cdot H_2O$ (%): C 48.65, H 4.42, N 12.29; Found: C 48.43, H 4.34, N 12.20.

3-Yb (deuterated)**:**

$Na_3[Yb(dpa)_3] \cdot 6\ H_2O$ (28 mg, 32 mmol) and $[Cr(ddpd)_2]Cl_3$ (24 mg, 32 mmol) in CD_3OD (2 mL), precipitation with $[D_8]$-^{i}PrOH → Yield: 33 mg (29 mmol, 79 %).

Anal. Calcd. for: $C_{55}H_{43}CrN_{13}O_{12}Yb \cdot 2\ CD_3OD \cdot 5\ H_2O$ (%): C 46.72, H 4.75, N 12.43; Found:. C 46.21, H 4.55, N 12.21.

3-Lu (deuterated)**:**

$Na_3[Lu(dpa)_3] \cdot 8\ H_2O$ (40 mg, 45 mmol); $[Cr(ddpd)_2]Cl_3$ (34 mg, 45 mmol) in CD_3OD (3 mL), precipitation with $[D_8]$-^{i}PrOH → Yield: 35 mg (26 mmol, 57 %).

Anal. Calcd. for: $C_{55}H_{43}CrN_{13}O_{12}Lu \cdot 2\ CD_3OD \cdot 5\ H_2O$ (%): C 46.66, H 4.74, N 12.41; Found: C 46.38, H 4.77, N 12.89.

2. X-ray Single Crystal Structure Analysis

X-ray data were collected with a Bruker Smart APEX II diffractometer with graphite-monochromated Mo K_{α} radiation or a Bruker APEX II Duo diffractometer with a Mo IμS microfocus tube and a TRIUMPH monochromator. The programs used were Bruker's APEX2 v2011.8-0, including SADABS for absorption correction, SAINT for data reduction and SHELXS for structure solution, as well as the WinGX suite of programs version 1.70.01 or the GUI ShelXle, including SHELXL for structure refinement.[S3] Deposition numbers CCDC 2003420-2003421 contain the supplementary crystallographic data for this paper. These data are provided free of charge by the joint Cambridge Crystallographic Data Centre (www.ccdc.cam.ac.uk/structures).

Table S1. Selected crystallographic data for **3-Yb** and **3-Lu**.

	**3-Yb**	**3-Lu**
Empirical formula	$C_{63}H_{71}CrN_{12}O_{19}Yb$	$C_{62}H_{68}CrLuN_{12}O_{18}$
M_r [g mol^{-1}]	1525.35	1496.25
Crystal appearance	orange prisms	yellow prisms
T [K]	100(2)	100(2)
λ [Å]	0.71073 (Mo K_{α})	0.71073 (Mo K_{α})
Crystal system	monoclinic	monoclinic
Space group	$P2_1/n$	$P2_1/n$
Z	4	4
a [Å]	16.1841(5)	16.1542(3)
b [Å]	23.2508(6)	23.3848(4)
c [Å]	17.6628(4)	17.6738(3)
β [°]	91.979(2)	91.789(1)
V [Å^3]	6642.4(3)	6673.2(2)
D_{calc} [g cm^{-3}]	1.525	1.489
μ [mm^{-1}]	1.644	1.712
F(000)	3116	3052.0
Crystal size [mm × mm × mm]	0.17 × 0.15 × 0.14	0.22 × 0.20 × 0.18
θ range [°]	1.45-27.56	1.45-29.18
Limitting indices	$-21 \le h \le 16$	$-21 \le h \le 22$
	$-25 \le k \le 30$	$-32 \le k \le 31$
	$-21 \le l \le 21$	$-24 \le l \le 24$
Collected refl.	41041	140668
Independent refl.	15071	17940
Absorption corr.	multi-scan	multi-scan
Transm. (max., min.)	0.746, 0.651	0.746, 0.650
Parameters/restraints	878/1	878/2
R_1 [$I > 2\sigma(I)$][a]	0.0549	0.0540
wR_2 [$I > 2\sigma(I)$][b]	0.1148	0.1268
R_1 (all data)[a]	0.0871	0.0592
wR_2 (all data)[b]	0.1290	0.1302
GOF on F^2	1.024	1.251
$\Delta\rho_{max,min}$ [e Å^{-3}]	1.621 /-1.630	3.134 / -3.250

[a] R_1 factor definition: $R_1 = \Sigma (||F_0| - |F_c||) / \Sigma |F_0|$
[b] wR_2 factor definition: $wR_2 = [\Sigma w(F_0^2 - F_c^2)^2 / \Sigma w(F_0^2)]^{1/2}$. Weighting scheme: $w = 1 / [\sigma^2(F_0)^2 + (np)^2]$, $p = [F_0^2 + 2 F_c^2] / 3$

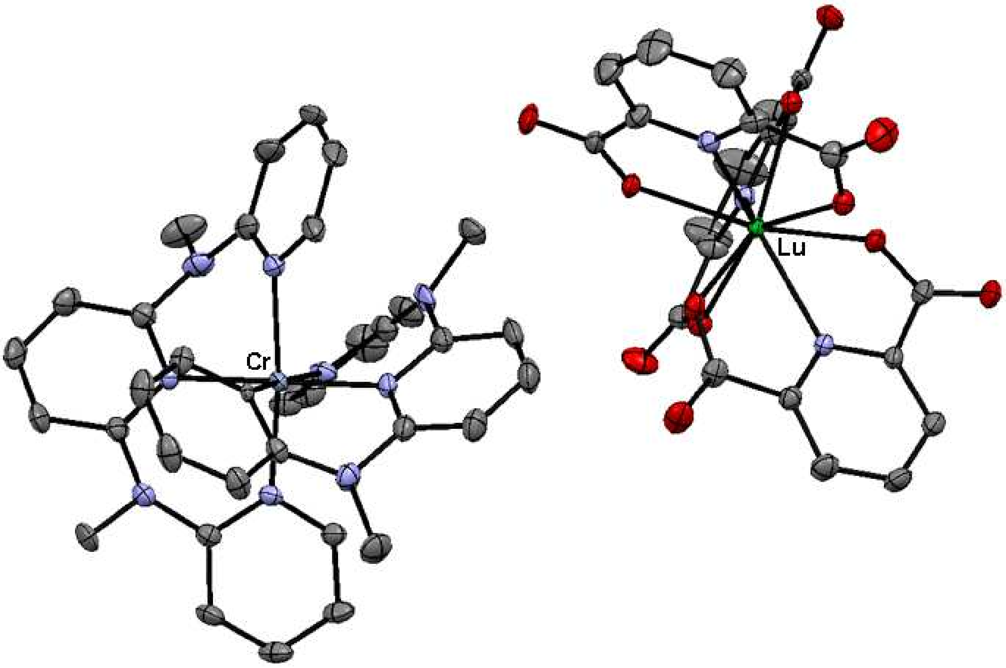

Figure S1. Thermal ellipsoid plot for **3-Lu** (Ortep 3 for Windows,[S4] 50% probability level). The lattice solvent molecules and hydrogen atoms in the complexes are omitted for clarity.

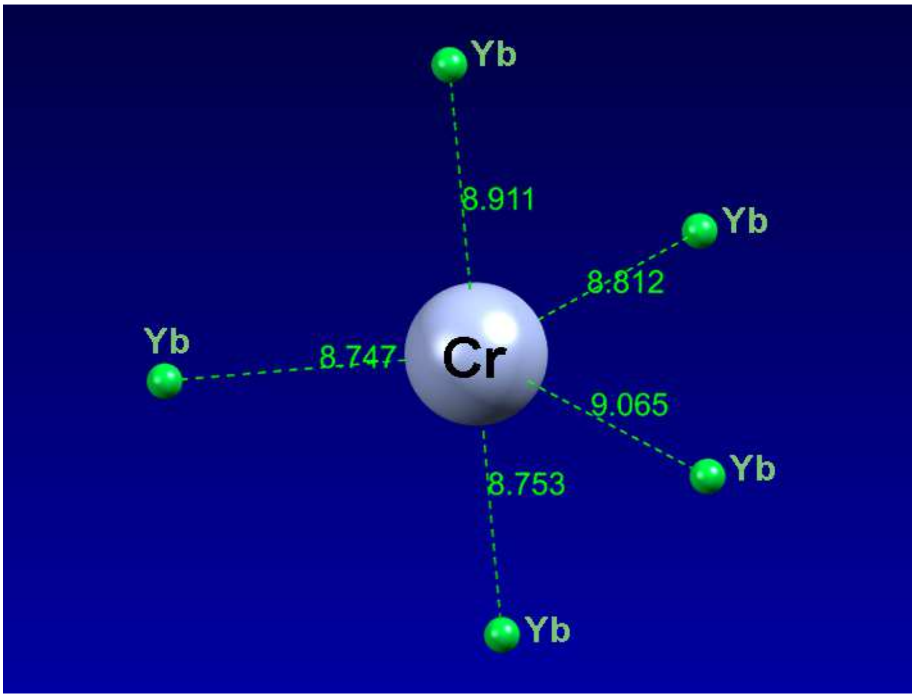

Figure S2. Distances Cr-Yb (Å) in the closest shell of Yb^{3+} sensitizers around each Cr^{3+} center in the solid state structure of **3-Yb**.

3. Luminescence Measurements

3.1 Instruments

The measurements were performed on different instruments:

1) For photophysical measurements under 435 nm excitation, two different spectrofluorometers were applied:

a) A Horiba Fluorolog-3 spectrofluorometer equipped with a 450-W Xenon lamp was used for steady-state measurements. The emitted light was detected at an angle 90° to the excitation light using either a Hamamatsu R2658P PMT detector (300 nm < λ_{em} < 1010 nm) or by a Hamamatsu H10330-75 PMT detector (950 nm < λ_{em} < 1700 nm). Spectral selection in the excitation channel was accomplished by a DFX double monochromator (gratings: 1200 grooves/mm, 330 nm blaze) and in the emission channel by a spectrograph iHR550 (single grating: 1200 grooves/mm, 500 nm blaze) in the visible/NIR spectral region (λ_{em} < 1010 nm) and a spectrograph iHR320 (single grating: 600 grooves/mm, 1000 nm blaze) in the NIR spectral region (λ_{em} > 950 nm). Spectral correction of the emission spectra was performed with a correction curve implemented by the instrument manufacturer. The luminescence decay kinetics were determined at 298 K with a pulsed xenon flash lamp (pulse width of ca.1.5 µs FWHM). The analysis of the luminescence decay kinetics (deconvolution, statistical parameters, etc.) was performed using the software package DAS from Horiba.

b) A calibrated spectrofluorometer FSP 920 from Edinburgh Instruments was equipped with different excitation light sources and detectors covering a wavelength region of about 300 to 1700 nm.[S5] Luminescence decay curves were also obtained at 298 K with spectrofluorometer FSP 920; yet in this case a µs xenon flashlamp (100 Hz, pulse width ca. 2.4 µs FWHM) and a Hamamatsu R2658P PMT detector were used. The luminescence decays were analyzed by fitting the obtained decay curves with the software FAST (Fluorescence Analysis Software Technology, Edinburgh Instruments Ltd.).

c) The luminescence quantum yields of the solid samples were determined by an absolute method using the commercial integrating sphere setup Quantaurus-QY C11347-11 from Hamamatsu[S6] using an excitation wavelength of 435 nm (direct excitation of the Cr^{3+} complex). For measurements of these solid-state samples,

miniaturized Petri dishes made from quartz from the instrument manufacturer were used.

2) For upconversion luminescence measurements, a calibrated photoluminescence spectrofluorometer FLS 980 from Edinburgh Instruments was used. The FLS 980 spectrofluorometer is equipped with an ca. 8-W 976-nm laser diode (Roithner Lasertechnik GmbH, Vienna, Austria), which can be used in a continuous wave (cw) or pulsed mode. The excitation power of the laser at the sample position was previously characterized with a power meter (Newport 841-PE Powermeter). By adjusting the current of the laser, laser power ranges from 0.02 to 9.07 W. As the samples were sealed between two glass slides and measured in a sealed cuvette (4 x 10 mm), reflection of excitation source must be considered. The power losses caused by laser reflections on the glass/quartz surfaces were determined to be ca. 11% by undergoing five separate measurements with different laser power.[S7] To gauge the excitation power density, a digital microscope was used to determine the laser spot size using ImageJ. The magnification factor of the microscope was calibrated with a standard calibration target (R1DS1N, USAF 1951 pattern, Thorlabs). This yielded an average spot size of about $6.6 \cdot 10^{-3}$ cm^2. The excitation power density was then calculated with respect to each laser power used in this study, which ranged from ca. 50 to 1230 $W{\cdot}cm^{-2}$. UC lifetime measurements were performed with pulsed 976-nm laser excitation (100 Hz, pulse width ca. 200 µs) with multi-channel scaling mode (MCS). A 645 nm long pass filter was applied to exclude the second order emission from the laser at 490 nm.

3.2 Steady-State and Time-Resolved Luminescence Measurements

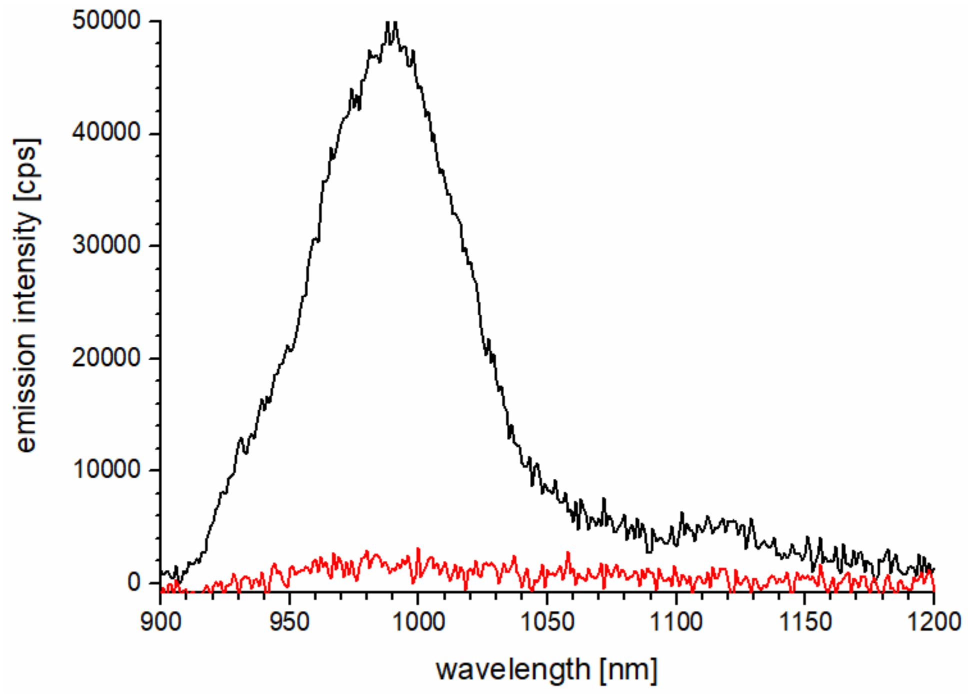

Figure S3. Steady-state emission spectra for **1-Yb** (solid state, 298 K, air) after excitation at 290 nm (black) and 435 nm (red).

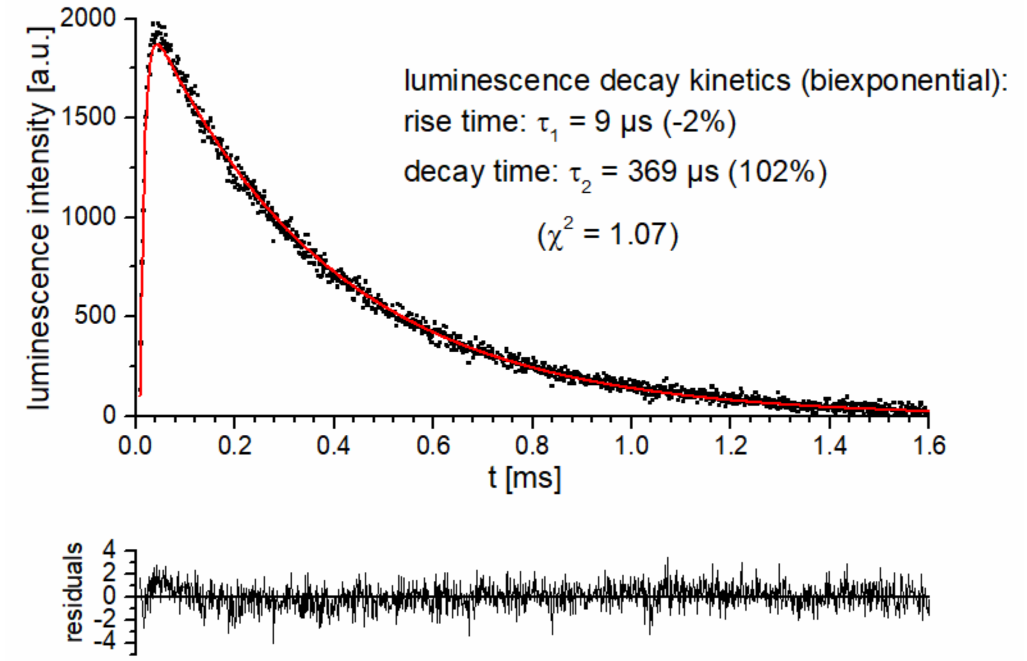

Figure S4. Luminescence decay profile (black scatter plot) obtained at 980 nm ($^2F_{5/2} \rightarrow {}^2F_{7/2}$ transition of Yb^{3+}) in **3-Yb** resulting for excitation at the Cr^{3+} absorption band at 435 nm ($^4A_2 \rightarrow {}^4T_2$ transition) at T = 298 K in air. The biexponential fit (red solid line) was obtained using a single rise time of 9 μs and a single decay component with a decay time of 369 μs. The residual shown below underlines the suitability of the fit.

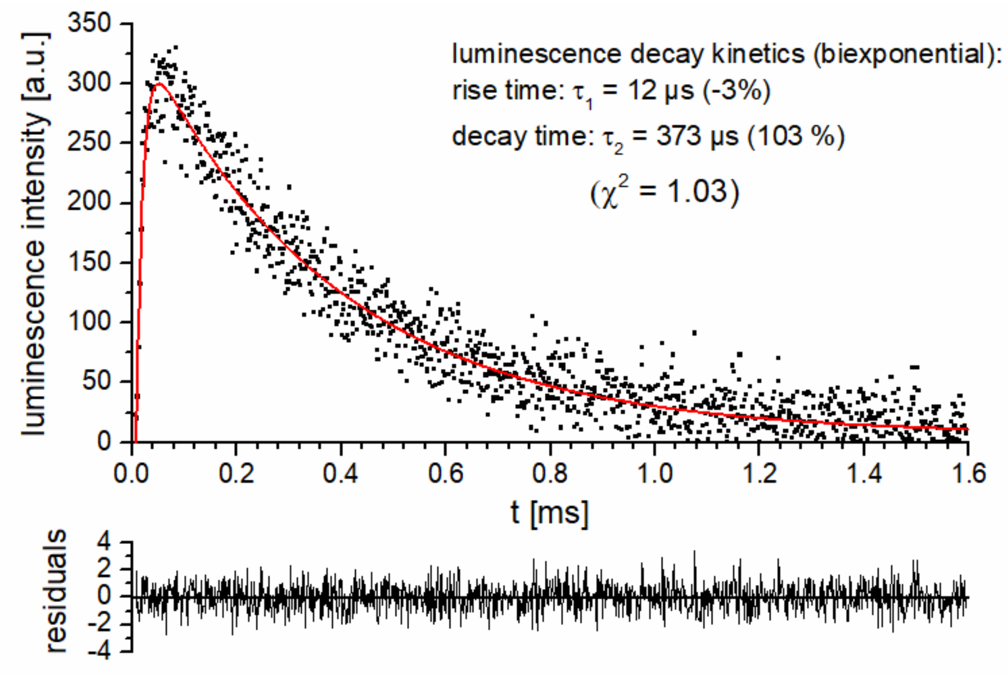

Figure S5. Luminescence decay profile (black scatter plot) for the transition $^2F_{5/2} \rightarrow {}^2F_{7/2}$ of Yb^{3+} (λ_{em} = 980 nm) in **3-Yb** (deuterated) after selective excitation into the Cr^{3+} absorption band $^4A_2 \rightarrow {}^4T_2$ (λ_{exc} = 435 nm) at T = 298 K in air – Biexponential fit (red solid line) with one rise time and one decay time component.

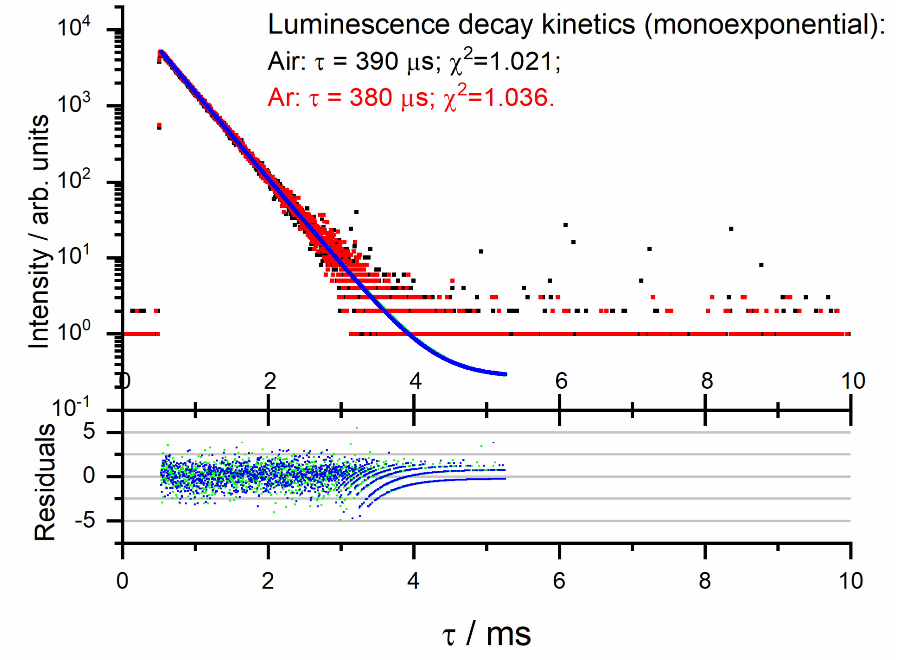

Figure S6. Luminescence decay profile under air- (black scatter plot) and Ar-saturation (red scatter plot) for the transition $^2E/^2T_1 \rightarrow {}^4A_2$ of Cr^{3+} (λ_{em} = 783 nm) in **3-Yb** after selective excitation into the Cr^{3+} absorption band $^4A_2 \rightarrow {}^4T_2$ (λ_{exc} = 435 nm) at T = 298 K – Monoexponential fits of the decay in air (green solid line) and in Ar (blue solid line).

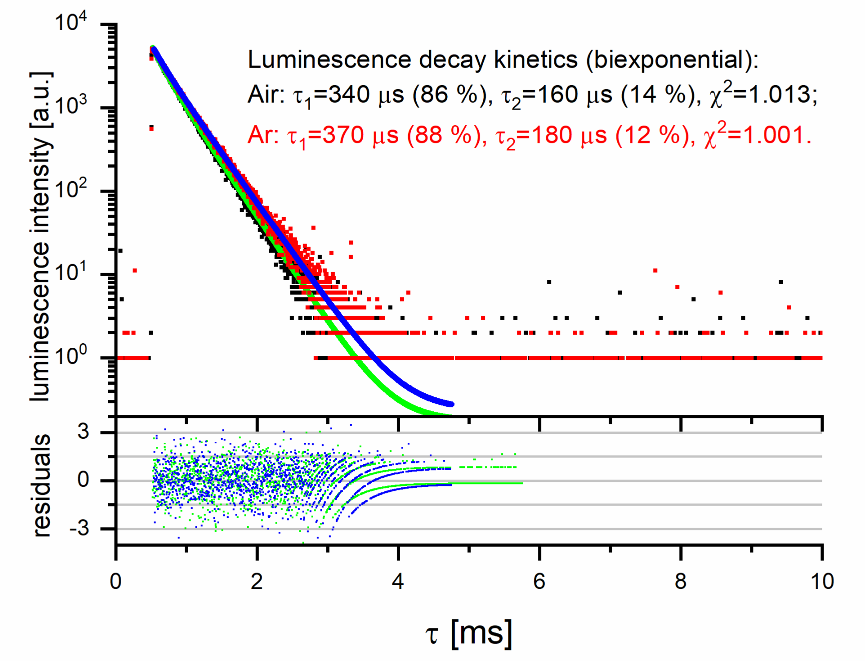

Figure S7. Luminescence decay profile under air- (black scatter plot) and Ar-saturation (red scatter plot) for the transition $^2E/^2T_1 \rightarrow {}^4A_2$ of Cr^{3+} (λ_{em} = 783 nm) in **3-Yb** (deuterated) after selective excitation into the Cr^{3+} absorption band $^4A_2 \rightarrow {}^4T_2$ (λ_{exc} = 435 nm) at T = 298 K – Biexponential fit of the decay in air (green solid line) and in Ar (blue solid line).

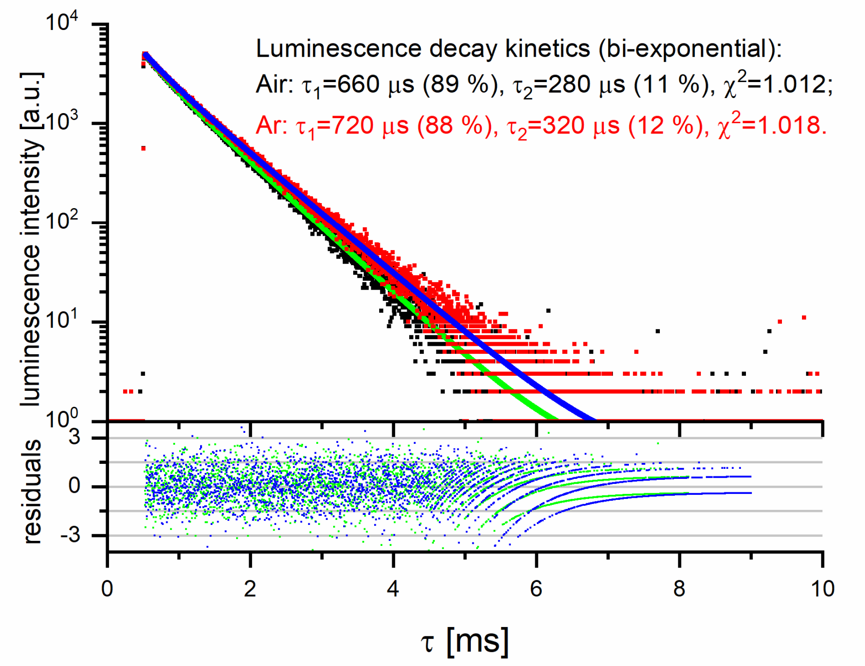

Figure S8. Luminescence decay profile under air- (black scatter plot) and Ar-saturation (red scatter plot) for the transition $^2E/^2T_1 \rightarrow {}^4A_2$ of Cr^{3+} (λ_{em} = 783 nm) in **3-Lu** after selective excitation into the Cr^{3+} absorption band $^4A_2 \rightarrow {}^4T_2$ (λ_{exc} = 435 nm) at T = 298 K – Biexponential fit of the decay in air (green solid line) and in Ar (blue solid line).

3.3 Upconversion Measurements

Within the optical UC emission spectrum, unfortunately, several spectral components cluttered the spectrum for peak interpretation. Thus, a mathematical approach was applied to flag and remove the undesired features from the spectra. Under the current experimental condition, the residual irradiation stemming from the excitation laser at 976 nm was removed from the spectra. The data points corresponding to the range 810 to 820 nm were fitted with a Gaussian function, where the center of this kernel function was constrained at 976 nm, to mimic the residual laser irradiation. Thereafter, this fitted model was applied across the entire measurement range, *i.e*. 700-840 nm, resulting in processed spectra containing only emission signals originating from the samples. Further, the spectra after removing the laser component were decomposed by:

$$f(\nu) = \sum_i A_i \cdot e^{-\left(\frac{\nu - \nu_{0,i}}{\sigma_i}\right)^2} + C \qquad \text{eq. S1}$$

where $f(\nu)$ is the fitted model, ν is the independent variable in the unit of cm^{-1}, A_i is the amplitude of spectral component i, $\nu_{0,i}$ is the peak center of component i, σ_i is the corresponding peak width of component i, and C is the bias constant. Specifically, the wavelength was converted to wavenumber in order to reflect the Gaussian nature of the fluorescence. In this scenario, three Gaussian kernels were used to interpret all spectra, in which the spectral components of interest fell onto the ones centered at 745 and 777 nm. All calculations were performed with Matlab 2017a.

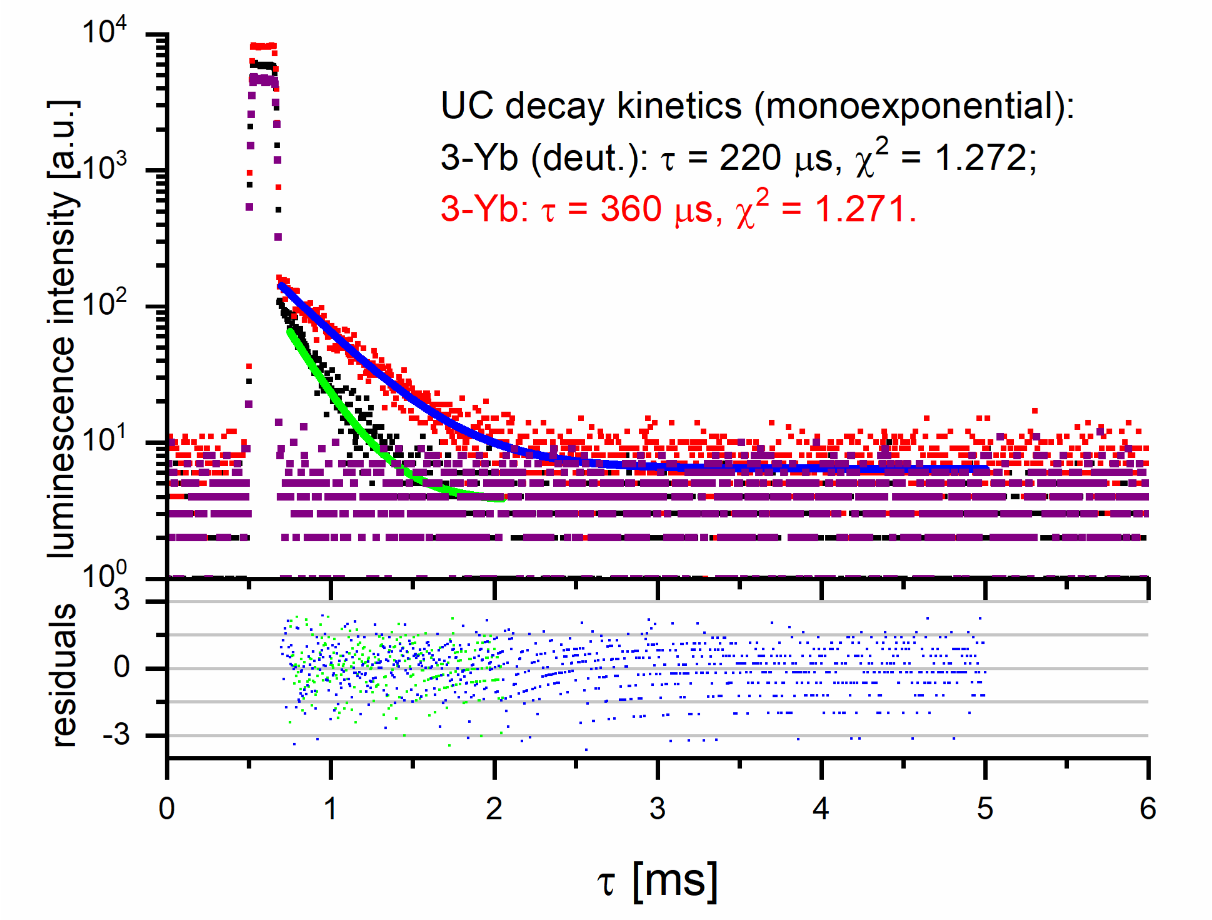

Figure S9. UC Luminescence decay profile of deuterated **3-Yb** (black scatter plot), **3--Yb** (red scatter plot) and **3-Lu** (purple scatter plot) for the transition $^2E/^2T_1 \rightarrow {}^4A_2$ of Cr^{3+} (λ_{em} = 778 nm) under selective excitation into the Yb^{3+} absorption band $^2F_{7/2} \rightarrow {}^2F_{5/2}$ (λ_{exc} = 976 nm) at T = 298 K – Monoexponential fit of the decay for deuterated **3-Yb** (green solid line) and of **3-Yb** (blue solid line).

4. References

[S1] G. Kervern, A. D’Aléo, L. Toupet, O. Maury, L. Emsley, G. Pintacuda, *Angew. Chem. Int. Ed.* **2009**, *48*, 3082.

[S2] A. Breivogel, C. Förster, K. Heinze, *Inorg. Chem.* **2010**, *49*, 7052.

[S3] a) L. J. Farrugia, *J. Appl. Crystallogr.* **1999**, *32*, 837; b) C. B. Hübschle, G. M. Sheldrick, B. Dittrich, *J. Appl. Crystallogr.* **2011**, *44*, 1281; c) G. M: Sheldrick, *Acta Cryst., Sect. A* **2008**, *64*, 112; d) *Bruker AXS Inc. Madison, Wisconsin, USA,* **2007**; e) G. M. Sheldrick, *SADABS, University of Göttingen, Germany,* **2008**.

[S4] L. J. Farrugia, *J. Appl. Crystallogr.* **1997**, *30*, 565.

[S5] S. Hatami, C. Würth, M Kaiser, S. Leubner, S. Gabriel, L. Bahrig, V. Lesnyak, J. Pauli, N. Gaponik, A. Eychmüller, U. Resch-Genger, *Nanoscale* **2015**, *7*, 133.

[S6] a) S. Würth, D. Geißler, T. Behnke, M. Kaiser, U. Resch-Genger, *Anal. Bioanal. Chem.* **2015**, *407*, 59; b) C. Würth, M. G. González, R. Niessner, U. Panne, C. Haisch, U. Resch-Genger, *Talanta* **2012**, *90*, 30; c) C. Würth, J. Pauli, C. Lochmann, M. Spieles, U. Resch-Genger, *Anal. Chem.* **2012**, *84*, 1345.

[S7] S. Gharaati, C. Wang, C. Förster, F. Weigert, U. Resch-Genger, K. Heinze, *Chem. Eur. J.* **2020**, 26, 1003.
